# Deep learning for non-parameterized MEMS structural design

**DOI:** 10.1038/s41378-022-00432-9

**Published:** 2022-08-29

**Authors:** Ruiqi Guo, Fanping Sui, Wei Yue, Zekai Wang, Sedat Pala, Kunying Li, Renxiao Xu, Liwei Lin

**Affiliations:** 1 Department of Mechanical Engineering, University of California, Berkeley, CA 94720 USA; 2 School of Computer Science, Wuhan University, Wuhan, 430072 China; 3 Department of Engineering Mechanics, Tsinghua University, Beijing, 100084 China

**Keywords:** Engineering, Materials science, Electrical and electronic engineering

## Abstract

The geometric designs of MEMS devices can profoundly impact their physical properties and eventual performances. However, it is challenging for researchers to rationally consider a large number of possible designs, as it would be very time- and resource-consuming to study all these cases using numerical simulation. In this paper, we report the use of deep learning techniques to accelerate the MEMS design cycle by quickly and accurately predicting the physical properties of numerous design candidates with vastly different geometric features. Design candidates are represented in a nonparameterized, topologically unconstrained form using pixelated black-and-white images. After sufficient training, a deep neural network can quickly calculate the physical properties of interest with good accuracy without using conventional numerical tools such as finite element analysis. As an example, we apply our deep learning approach in the prediction of the modal frequency and quality factor of disk-shaped microscale resonators. With reasonable training, our deep learning neural network becomes a high-speed, high-accuracy calculator: it can identify the flexural mode frequency and the quality factor 4.6 × 10^3^ times and 2.6 × 10^4^ times faster, respectively, than conventional numerical simulation packages, with good accuracies of 98.8 ± 1.6% and 96.8 ± 3.1%, respectively. When simultaneously predicting the frequency and the quality factor, up to ~96.0% of the total computation time can be saved during the design process. The proposed technique can rapidly screen over thousands of design candidates and promotes experience-free and data-driven MEMS structural designs.

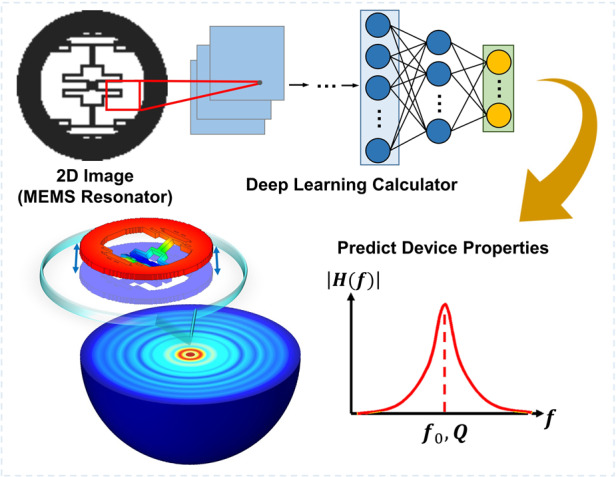

## Introduction

Over recent decades, machine learning (ML) has been considered an important innovation with prodigious success in industry^1^. One key aspect of ML is that it improves itself automatically by uncovering the critical relationship between raw inputs and final outputs from a given dataset. This self-updating nature of ML has benefited a broad range of interdisciplinary fields, such as robotics^2^, health informatics^3^, protein engineering^4^, statistical physics^5^, computational chemistry^6^, and material discoveries^7^. Modern ML technologies can be integrated with advances in mechanics to drive optimal design solutions in MEMS. In previous research, ML techniques have been successfully implemented to analyze device signals^8–10^ and to design device structures^11–14^. While the latter works have led to pioneering results for data-driven MEMS design, they generally require a high level of prior knowledge in the field. In these works, the basic design topology is first determined, and the detailed structural parameters are then optimized using ML algorithms. In this study, we introduce a data-driven nonparameterized design approach as an important alternative. The nonparameterized design method constructs target structures voxel-by-voxel from scratch, without constraints in given topologies^15–22^. Instead, when given a few design variables, such as the overall size and material properties, a very large number of design combinations can be created. Historically, this approach was computationally expensive for traditional ML, and the generated innumerable datasets resulted in complexities during analysis. The deep learning (DL) technique used in this study provides a good solution, as it can effectively learn the hidden patterns from a large number of datasets. The DL approach builds predictive models with multiple levels of simple but nonlinear modules that transform the representations at each level to a slightly higher level of representations. With a sufficient number of layers, very complex hidden patterns can be determined by the model^23^.

Using DL models, we have built an automated system framework applicable for experience-free, nonparameterized design of many categories of MEMS devices. In this work, we present MEMS resonators as an example. MEMS resonators have been intensively studied in the past for a wide range of applications, such as timing references, filters in wireless communication systems, and sensing elements in various modules^24^. Two important properties of a MEMS resonator are (1) the resonant modes/frequencies and (2) the quality (simply abbreviated as “Q”) factor. While a resonator has an infinite number of resonant modes, only a limited number of them have practical usage, such as the flexural mode^25^, bulk mode^26^ and wine-glass mode^27^. For many applications, a critical goal for resonator structural design is reducing the energy loss of a chosen mode (or, in other words, enhancing the Q-factor of that mode) to improve the sensitivity, resolution, and accuracy of the device^28^. Finding the right geometric structures is crucial in achieving the desirable resonant mode and frequency and a high Q-factor^29–31^. However, this search for the right design was a very challenging and time-consuming process for human intuition and numerical analysis. Our work aims to address this problem.

In this work, the geometries of disk-shaped MEMS resonators are represented with binary pixelated images. The two physical properties of interest are the resonant frequencies f and the Q-factor due to anchor loss Q_anchor_ (one iconic damping mechanism for microresonators, in addition to viscous and material damping)^26,29,32,33^. The computational results (f and Q_anchor_) obtained from carefully performed finite element analyses (FEA) following validated procedures are considered the ground truth^34–37^ and are used to label the images corresponding to each geometric configuration before the training of our DL model. After being trained by tens of thousands of samples, the DL model can accurately predict both f and Q_anchor_ with good accuracy, such that new candidate geometric configurations can be considered without even performing FEA on them. The DL models can predict the required physical properties of one design in 8.9 × 10^−3^ s. Since the forward computation of the DL models can be orders of magnitude (~2.6 × 10^4^ times) faster than FEA simulations, the DL models are used as high-speed surrogate calculators to remarkably reduce the time costs of the design process. We expect that the proposed method can also be extended beyond resonators and contribute to the design process of many categories of MEMS devices.

## Methods

### System architecture

**Fig. 1:**
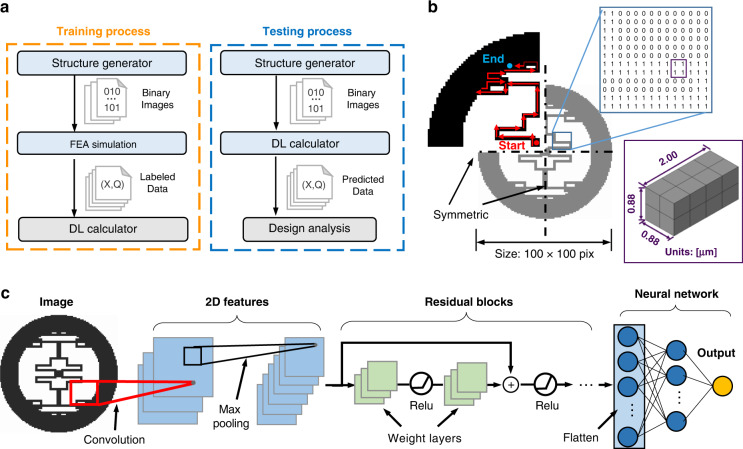
System components of the DL-assisted nonparameterized MEMS design. **a** The major components for the proposed system architecture, which include the training process and the testing process. **b** An example disk resonator pattern generated from Brownian motion, followed by folding along the two axes of symmetry. The pattern is represented as a 100 × 100 pixel matrix, where 0 is a void element and 1 is a solid element. Inset: representative finite element mesh for a 2 × 2 solid region. **c** The structure of the deep residual neural network model used in this work, featuring input images, 2D features, residual blocks, the neural network, and the output target.

The major components of the proposed system are illustrated in Fig. 1a, including the training process and the testing process. In the training process, a structure generator creates binary images representing the resonator structures, while the physical properties (f and Q_anchor_) of the corresponding geometries are labeled with FEA results. The DL model is trained using abundant labeled samples (referred to as “training samples”) in many epochs to form a DL calculator with good accuracy. In the testing process, the structure generator passes new samples (referred to as “testing samples”) to the DL calculator, and the DL calculator predicts the physical properties of these samples that have never appeared in the training process. After enough training, the DL calculator can accurately analyze the physics of candidate designs and help select good designs without the need for additional numerical simulations.

In this work, the resonators are made of polysilicon with density ρ = 2.3 × 10^3^ kg/m^3^, Young’s modulus E = 150 GPa, and Poisson’s ratio *v* = 0.29. For each disk resonator configuration, the diameters of the inner and outer rings, the diameter of the central anchor stem, and the thicknesses of the disk layer and the anchor stem are fixed parameters, as shown in Fig. S1. An example disk resonator pattern is shown in Fig. 1b as a 100 × 100 binary matrix, where 0 represents a void element and 1 represents a solid element. For the structural layer, each voxel is 0.44 µm in width and length and 0.5 µm in depth. This binary representation of the geometries is the key to achieving nonparameterized design. The agent is defined as a 2 × 2 × 4 solid element region that can move randomly stepwise along the four cardinal directions within one quadrant of the design domain. The trajectory of the Brownian-like motion for the agent always begins from the start point near the center anchor stem and stops at the endpoint near the inner annulus of the resonator. As another geometric constraint, the total area covered by the trajectory can be assigned a programmable value. By folding the trajectories along the two axes of symmetry, the agent connects the anchor stem and the outer annulus of the resonator. As such, the nonparameterized and pixelated geometric configurations of the resonator are formulated.

The physical properties of the resonator structures can be predicted with state-of-the-art DL models such as a residual neural network (ResNet)^38^, dense convolutional network (DenseNet)^39^, and EfficientNet^40^. The detailed modeling settings based on the PyTorch^41^ API of the three different DL models are described in Supplementary Note S1. Figure 1c presents the structure of a customized ResNet, which is the DL model eventually selected in this study. The model structure includes five basic components: the input image, 2D features, residual blocks, the neural network, and the output targets. The 2D feature maps are generated via matrix multiplication between the original input image and the convolution kernels to capture the influence of nearby pixels. The max pooling layer selects the maximum of feature maps as the inputs to subsequent layers. ResNet skips the training of a few layers by using residual blocks to solve the degradation problem of neural networks. The 2D vectors are then flattened into a 1D vector as the neural network input, while the fully connected layer applies a linear transformation to the input vector through a weighted matrix. The number of final output neuron(s), representing the physical properties of a MEMS design, can be either single or multiple.

### Modal frequency and anchor loss simulation

**Fig. 2:**
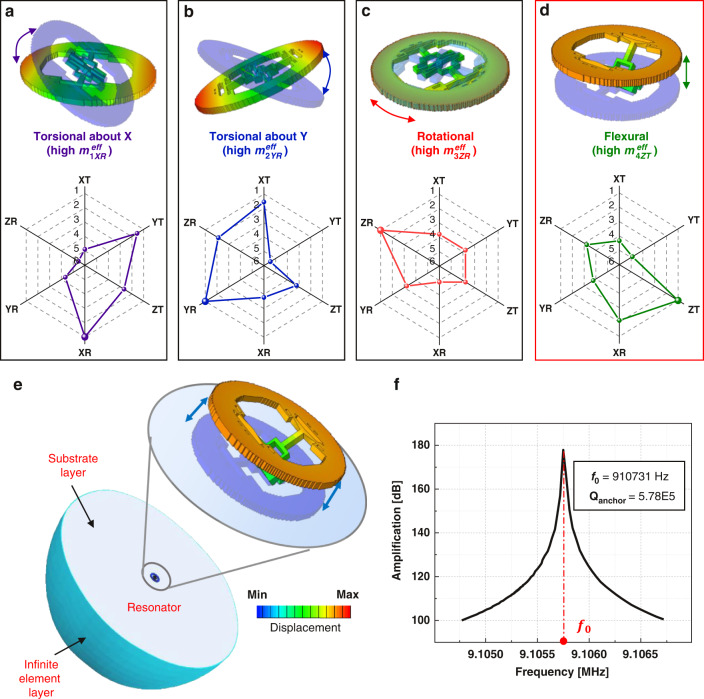
Computational methods for mode frequency identification and Q_anchor_ calculation. **a**–**d** The mode shapes (top row) and ranking distribution of directional effective mass components (bottom row) of the disk resonator in its first four modes. The rankings are ascending with the given $m_{\alpha i}^{{{{\mathrm{eff}}}}}$ among the 10 earliest appearing modes. **e** Schematic of the finite element simulation to estimate Q_anchor_ using the infinite element method. Different colors on the resonator structures in a-e represent normalized out-of-plane displacement magnitudes in the mode shapes. **f** The frequency-amplitude response of the sample resonator structure.

In this study, extensive FEA is conducted to generate results that are considered the “ground truth” in the training and validation processes. Two types of FEA are performed, namely, (1) natural frequency analysis for identifying the vibrational mode of interest^42^ and (2) complex frequency analysis for extracting Q_anchor_. As detailed in Supplementary Note S2, the natural frequency analysis yields the ideal, undamped frequency (real eigenvalue), the mode shape and the effective mass corresponding to each vibrational mode. For a certain mode *α*, the vibration motion of the resonator can be projected into six directions (*j*), namely, translation along the *X*, *Y*, or *Z* axis (*i* = XT, YT, or ZT) and rotation about the *X*, *Y*, or *Z* axis (*i* = XR, YR, or ZR). As described in Supplementary Note S2, the effective mass from FEA outputs m^eff^ is a two-dimensional tensor, with one component $m_{\alpha i}^{{{{\mathrm{eff}}}}}$ describing the amount of mass in the system participating in motions along a certain direction *j* in a vibration mode *α*. Using this critical tensor m^eff^, a vibrational mode *α* can be automatically identified by comparing the relative values of each component $m_{\alpha i}^{{{{\mathrm{eff}}}}}$ in the row, instead of through tedious human visual inspections of the mode shapes. In this manner, the corresponding vibrational modes at calculated natural frequencies can be distinctly identified and labeled, which significantly reduces the time and labor consumption during the data training or testing preparation process when dealing with thousands of samples. As an example, Fig. 2a–d show the mode shapes (upper row) and the distributions of effective mass components (lower row) corresponding to the first four vibrational modes of one resonator. The four modes are the torsional mode about the *X* axis (*α* = 1), torsional mode about the *Y* axis (*α* = 2), in-plane spinning mode (*α* = 3), and out-of-plane flexural mode (*α* = 4). The radar charts show the rankings of the six directional effective mass components in these modes, from the highest (ranking = 1, outermost) to lowest (ranking = 6, innermost). As expected, the corresponding rotational components ($m_{{{{\mathrm{1XR}}}}}^{{{{\mathrm{eff}}}}}$, $m_{{{{\mathrm{2YR}}}}}^{{{{\mathrm{eff}}}}}$, $m_{{{{\mathrm{3ZR}}}}}^{{{{\mathrm{eff}}}}}$) are ranked first in the two torsional modes and the spinning mode, while the Z-direction translation ($m_{{{{\mathrm{4ZT}}}}}^{{{{\mathrm{eff}}}}}$) is the highest in the flexural mode. Utilizing this information, we can conveniently distinguish the mode of interest (the “flexural mode”, *α* = 4) from all the modes computed through FEA and obtain the corresponding natural frequency ω_flex_.

The computed natural frequency for the flexural mode, ω_flex_, is then used in subsequent complex frequency analysis studies, where 0.98ω_flex_ and 1.02ω_flex_ define the lower and upper bounds for frequency searching. The ±2% range accounts for the difference in value between damped and undamped natural frequencies. As shown in Fig. 2e, the bottom surface of the resonator’s anchor is attached to a sufficiently large hemispherical substrate (radius = 0.5 mm, 22.7 times larger than the resonator structure) in the complex frequency analysis. The substrate is enclosed by a layer of infinite elements for absorbing the transmitting elastic waves without reflection. With this FEA setup, we can calculate the complex-valued frequency for the flexural mode, $\omega _{{{{\mathrm{flex}}}}}^C$, and obtain the Q-factor due to anchor loss, ${{{\mathrm{Q}}}}_{{{{\mathrm{anchor}}}}} = \frac{{{{{\mathrm{Real}}}}\left[ {\omega _{{{{\mathrm{flex}}}}}^C} \right]}}{{{{{\mathrm{2Imag}}}}\left[ {\omega _{{{{\mathrm{flex}}}}}^C} \right]}}$, to label each sample. Figure 2f illustrates the frequency response of the representative resonator structure shown in Fig. 2a–e, featuring the peak at the damped natural frequency and the corresponding Q_anchor_ value. For the resonator structure shown in Fig. 2a–e, this complex frequency analysis step yields a damped natural frequency and Q_anchor_ of 910,731 Hz and 5.78 × 10^5^, respectively. These values are consistent with the frequency response shown in Fig. 2f obtained from a steady-state dynamics study.

## Results and discussions

### Dataset description and DL calculator interpretation

**Fig. 3:**
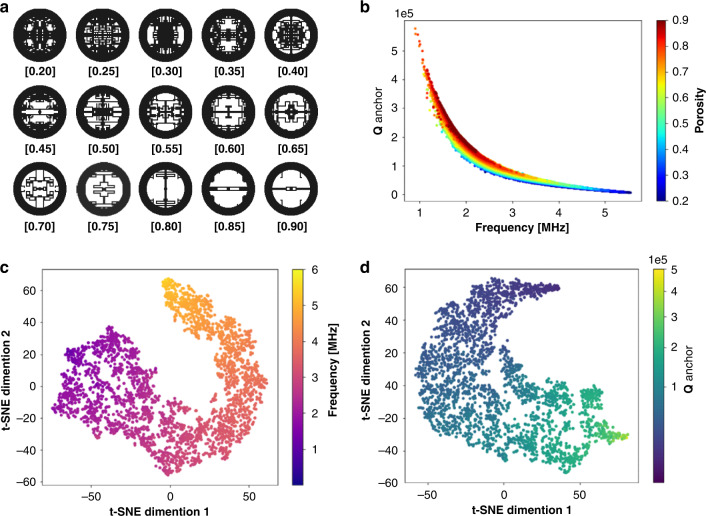
Visualized dataset and DL calculator interpretation. **a** Examples of generated resonator patterns with different porosity values. **b** Frequency vs. Q_anchor_ plot for 29,984 samples. Color denotes the porosity value of each sample. **c**, **d** Two-dimensional t-SNE analysis of hidden layer vectors, including frequency and Q_anchor_, from the DL calculator on the testing set.

The region between the anchor stem at the center and the outer annulus structure introduces a vast design space. To provide a sufficient number of training samples, 29,984 unduplicated resonator patterns are created. Patterns are shown in Fig. 3a as an example, in which the ratio of void elements versus the total pixel numbers in the design space is defined as “porosity” and labeled to the pattern. To provide a balanced representation of the design space with the dataset, equal numbers of samples are generated at approximately 15 levels of porosity values, which are evenly spaced from 0.2 to 0.9, with an interval of 0.05. The computation time for generating each structure input is as low as 1.2 s on average such that the proposed input configuration generation method has high throughput. Figure 3b illustrates the relationship between the resonant frequency, porosity and Q_anchor_ value of the samples. As can be observed from the dataset, the dominating trend is that the energy loss increases and the Q_anchor_ value decreases as the resonating frequency increases, which is typical for MEMS resonators^43–45^ and reveals the difficulty in achieving high Q_anchor_ and high frequency simultaneously. All obtained f-Q_anchor_ products are of the same order of magnitude, with an average value of (2.2 ± 1.0) × 10^11^. The correlations among porosity, frequency and Q_anchor_ are plotted in Fig. S2a, b. While as an overall trend, the resonant frequency and Q_anchor_ increase and decrease, respectively, with higher porosity, the wide spreads of data (almost ten times different) in these graphs suggest that we cannot oversimplify the dependency of resonator properties on geometric details to one on a single parameter, porosity. Therefore, we must utilize DL to comprehensively learn the geometric details from pixelated images and find more hidden patterns to accurately predict these physical properties.

In the data preparation process, ~90% of the dataset (26,985 samples, all with FEA results) is labeled and used as the training set, and the remaining ~10% (2999 samples) is used as the testing set. The two ResNet-based single-output DL calculators for frequency and Q_anchor_ predictions are trained separately using the training set and validated using the testing set. After the training process, both DL models are explained using the t-distributed stochastic neighbor embedding (t-SNE) method with the default scikitlearn^46^ settings, which visualizes high-dimensional vectors by assigning each data point a location in a two-dimensional map^47^. If the vectors share similar features, their corresponding locations are close to each other in the low-dimensional map. As each resonator pattern image is inputted into each DL calculator and the last layer hidden neuron values are calculated, a high-dimensional vector with a size of 2048 is obtained. For the predictions of the DL calculator on the testing set, the corresponding high-dimensional vectors are grouped together and visualized through t-SNE in Fig. 3c, d, where the data points are colorized with the FEA-calculated frequency labels (Fig. 3c) and Q_anchor_ labels (Fig. 3d). It can be observed that the frequency and Q_anchor_ values of the testing samples smoothly change from one end of the shape to the other end, indicating effective training of the DL networks, as testing samples with similar physical properties would indeed be neighboring points in the high-dimensional space. The same visualization approach is also applied to the training set samples in Fig. S3a, b, where the dominating trend matches well with results from the testing set. In addition, we find that data points colorized by porosity values Figs. S4 and S5 do not yield a smooth transition in color. This finding agrees with the results in Fig. S2 and further proves that the physical properties of a resonator would depend on the geometric details, not on just the porosity of the structure.

### Performance evaluation of the DL calculators

Three evaluation metrics are considered in this study to select a suitable DL model. (1) The forward calculation efficiency for predicting the target physical properties, as measured by the sample averaged testing time. (2) The computational time costs to obtain the DL calculators, as measured by the total model training (back propagation) time. (3) Regression accuracies of the target physical properties, evaluated at each sample point as ${{{\mathrm{Accuracy = 1 - }}}}\left| {\frac{{y_i - \hat y_i}}{{y_i}}} \right|$, where y_i_ is the ground-truth label value (from FEA) of sample i and $\hat y_i$ is the predicted value from the DL models. As shown in Fig. S6a, b, the ResNet50 models are approximately 3 and 2 times faster than the DenseNet201 and EfficientNetB4 alternatives for forward calculation. Fig. S6c, d shows that the total training time t increases linearly with respect to the sample number x, while the ResNet50 models are 1.8 and 1.7 times faster than those of DenseNet201 and EfficientNetB4 for the model training process. On the other hand, the differences in accuracies among the three models are within 1% when the total sample amount reaches 24,300 (Fig. S6e, f). Based on these results, ResNet50 is selected for the subsequent studies.

**Fig. 4:**
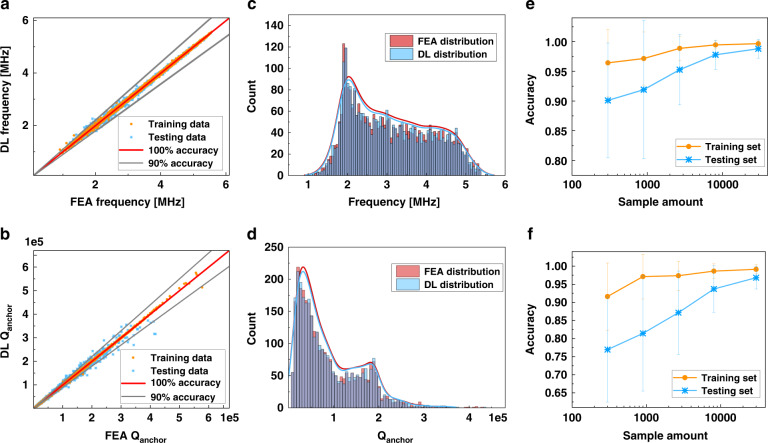
The regression performance evaluation for resonant frequency (top row) and Q_anchor_ (bottom row) of the DL calculators. **a**, **b** Comparisons between DL predictions and the FEA simulations for **a** frequency and **b** Q_anchor_. **c**, **d** Distributions of data in DL and FEA for the testing set in **c** frequency and **d** Q_anchor_. **e**, **f** Learning accuracy vs. number of samples in the dataset for **e** frequency and **f** Q_anchor_ studies. Error bars denote standard deviations.

Figure 4a, b show a comparison between the ResNet50-based single-output DL predictions and FEA simulations for frequency and Q_anchor_, respectively. Trained with 26,985 samples (90% of the total 29,984 samples), the highest average accuracies of the testing sets (2999 samples) are 98.8 ± 1.6% and 96.8 ± 3.1% for the frequency and Q_anchor_ regression, respectively. The learning curves for this experiment in Fig. S7a, b show the L1 loss versus training epochs, where both the training and the testing curves converge in the end. Figure 4c, d illustrate the sample distribution of data in the testing set from the DL model and from FEA. For both frequency and Q_anchor_, the DL and FEA distributions show good alignment. The frequency distribution could be viewed as a right-skewed distribution with peak density at ~2 MHz and a nearly even distribution in the range between 2.5 and 4.8 MHz. It is noteworthy that Q_anchor_ also shows a right-skewed distribution but with high kurtosis. This matches our expectation that most geometries provide low Q_anchor_ values and that geometric designs with exceptionally high Q values are rare and would require plenty of iteration efforts. Figure 4e, f shows how the regression accuracy changes with the number of samples for the frequency and Q_anchor_ predictions, respectively. Before the sample amount reaches 10,000, increasing the number of samples leads to obviously higher accuracy and lower standard deviation. The performance enhancement becomes less obvious with further added samples. Given that the average testing accuracy surpasses 95% for both frequency and Q_anchor_, we consider the sample amount to be sufficient at this point. These findings are also supported by the learning curves and DL vs. FEA comparisons performed on 300, 900, 2700, and 8100 samples in Figs. S8 and S9.

### DL calculators for design ranking and computation time cost reduction

To be applicable in the highly demanded field of high-Q-factor resonator designs, the DL calculator must be capable of recognizing whether one design is superior to another. During the experiment, the testing samples are first sorted in ascending order according to their DL predicted Q_anchor_ values and labeled by the predicted rankings, obtaining a natural number sequence <1, 2, …*n*>, where *n* is the total number of testing samples for ranking. Afterward, the testing samples are sorted in ascending order according to their actual FEA simulated Q_anchor_ values and the previously defined natural number sequence transforms to a new sequence X = <x_1_, x_2_, …, x_n_>. Here, we quantitatively evaluate the performance of our DL model in comparing vast different samples based on Q_anchor_ values using ranking accuracy (RA) as the metric, which is defined as

1 $$R{\rm{A}}=\frac{{\# \{ ({\rm{i}},{\rm{j}})|({x_i} - {x_j})({\rm{i}} - {\rm{j}})\, > \, 0,1 \le {\rm{i}},{\rm{j}} \le {\rm{n}}\} }}{{{\rm{n}}({\rm{n}} - 1)/2}}$$

**Fig. 5:**
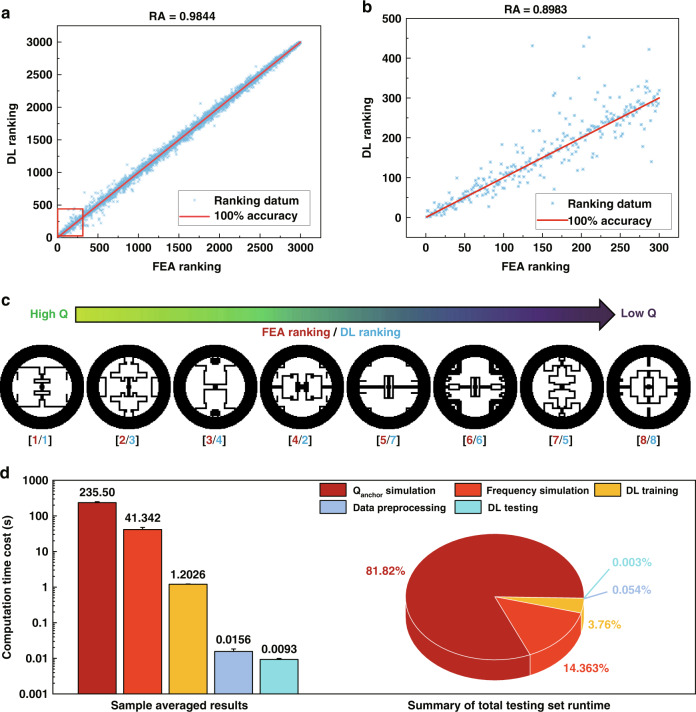
Ranking performance and computation cost. **a** Comparison of ranking obtained from the DL model and FEA simulation for the entire testing dataset. The red box features the top 10% of data in ranking. **b** Magnified view of the boxed region in **a**. **c** Patterns of the top eight designs based on high Q_anchor_ values in the testing dataset as calculated using the DL model. The numbers in the bottom square brackets are FEA Rankings/DL Rankings. **d** The histogram shows the computation time costs in terms of seconds for the FEA simulation for frequency and Q_anchor_, the data preprocessing, and the training and testing process for the double-output DL model. The pie chart shows the percentage of the computation time costs from the FEA simulations, data preprocessing, DL training and testing processes to analyze the frequency and Q_anchor_ of the MEMS disk resonators.

The symbol # denotes the number of elements that satisfies the described conditions. Each correctly predicted pair of unique integers x_i_ and x_j_ at the ith and jth positions of the sequence *X* should have (x_i–_x_j_)(i-j) > 0, and the corresponding (i,j) pair is recorded as a valid element. The total number of possible (i,j) pairs is n(n-1)/2; thus, RA indicates the portion of correctly ranked pairs within the total possible combinations. The overall *RA* value is 98.44%, as shown in Fig. 5a, when using the results of the testing set for evaluation. For the samples with the top 10% Q_anchor_ (considered to be “good designs”) in Fig. 5b, our DL calculator could still achieve a high *RA* value of 89.83% and can successfully find all the designs in the top 10%. To showcase our DL calculator’s capacity in identifying the desirable designs with exceptionally high Q_anchor_ (top 8 ranked, or equivalently top 0.3%), we show the specific geometries of these samples in Fig. 5c, along with their rankings using FEA (ground truth) and using our DL calculator. Even for this domain with very limited training data (due to the scarcity of high-Q structures), our DL calculator still performs remarkably, as it manages to find all eight best structures, correctly identifies three rankings (1st, 6th, and 8th), and yields a *RA* value of 82.14%.

After sufficient training, the DL calculators are not only accurate but also much faster than FEA in generating results for frequency and Q_anchor_. As shown in Fig. S10, FEA simulations take 41.3 ± 6.2 seconds and 235.5 ± 14.1 seconds on average to yield sample results for the frequency and Q_anchor_, respectively, whereas the single-output DL calculators only take 1.27 ± 0.013 seconds and 1.26 ± 0.008 seconds during the training process (back propagation) and 8.9 ± 0.67 × 10^−3^ s and 8.9 ± 0.48 × 10^−3^ s during the testing process (forward calculation). Therefore, the DL calculators can produce results for a given geometric structure 4.6 × 10^3^ times faster (in frequency calculation) and 2.6 × 10^4^ times faster (in Q_anchor_ calculation) than FEA for the single-output DL models. The above results indicate that while the time consumption for the FEA simulations can be very different for required physical properties, the averaged forward computation time costs for DL calculators are always negligible. The DL calculator can be trained to simultaneously predict two outputs (the frequency and Q_anchor_) to further accelerate the computation process. The testing regression accuracies for the frequency and Q_anchor_ of the double-output DL calculator are 98.6 ± 1.9% and 96.5 ± 4.1%, respectively, which are comparable with those of the single-output DL calculators, as shown in Fig. S11. In the histogram of Fig. 5d, the double-output DL calculator only takes 1.20 ± 0.024 s per sample to train the model and 9.3 ± 0.61 × 10^−3^ s per sample to simultaneously predict both outputs. The pie chart shows the break-up of time consumption in this study for this double-output model, where only 3.8% of time is spent on the training/testing of the DL calculators and the data preprocessing process (details described in Supplementary Note S1), while the remaining 96.2% of time is spent in FEA to generate high-quality label data for the training process. As such, for future design screening, a well-trained, double-output DL calculator can reduce the computation time by up to ~96.0% compared to purely FEA-based simulations.

## Conclusions

In this study, we applied a deep learning (DL) technique to calculate the physical properties of MEMS structures effectively and accurately. The geometries of numerous candidate designs were represented by pixelated binary images, which were then labeled by numerical simulation results and used in the training of neural networks. With sufficient training, the networks can learn the hidden patterns in the vast number of candidate geometries and calculate the physical properties (such as the frequency and quality factor of disk-shaped microresonators) quickly and accurately. The networks can also be used to rank thousands of candidate geometries based on a certain quantity of interest (e.g., quality factor) and guide researchers toward good designs. Not limited to the resonator design, the proposed approaches can be extended to other types of MEMS devices, such as microscale piezoelectric energy harvesters^48^ accelerometers, gyroscopes, etc. By combining the DL calculator with a DL designer in the future, the calculation results could directly guide the generation of new candidate geometries toward a desired design goal. Another possible future direction is to incorporate multilayer structural features in MEMS in our neural networks to apply our data-driven approach to even more complex MEMS devices. After choosing the desirable structural design, the data-driven approach could also be applied to predict and enhance the microfabrication process to account for the effects on the final device performance and reliability from parameters in key process steps (spin-coating, exposure, polysilicon and oxide deposition, etching, annealing, etc.), material surface morphology and imperfections, and anomalies during the process^49–52^.

## Supplementary information

Supporting Information
