# Supplementary material for: Deep learning for non-parameterized MEMS structural design
Source: Microsyst Nanoeng. 2022 Aug 29;8:91. doi: 10.1038/s41378-022-00432-9 (PMC9424241; doi:10.1038/s41378-022-00432-9)
Supplement: Supplementary file 1 — Supporting Information [file 41378_2022_432_MOESM1_ESM.docx]

**Supporting Information for**

**Deep Learning for Non-Parameterized MEMS Structural Design**

**Supplementary Note S1: Data Preprocessing and DL Modeling Details**

The pre-processing of data in this study includes two major steps: 1. encoding geometrical images and corresponding physical properties (frequency, $\text{Q}_{\text{anchor}}$) to the hard drive as the shelf dictionary objects. And 2. sending shelf dictionary objects to the computer RAM as data loaders for DL model training. The first step is carried out only once during the entire study and the second step is carried out each time before the model training starts. In the first step, there are three time consuming parts: python package importing (2.17 ± 0.31 s), data files loading (379.44 ± 83.73 s), and shelf dictionary objects saving (42.83 ± 2.98 s). In the second step, there are also three parts: DL module importing (6.06 ± 0.42 s), shelf dictionary objects loading (22.26 ± 0.35 s), image resizing and dataloader creation (15.94 ± 2.74 s). The total computation time cost for data preprocessing is 468.69 ± 82.3 s across different experiments.

The deep learning (DL) model architectures in this work are from the ResNet50, EfficientNetB4 and DenseNet201 that have comparable parameter sizes and sufficiently reported image recognition accuracies based on the other datasets^40^. They are adopted from the torchvision.models subpackage within the PyTorch platform of the same version for fair comparison. In the model training process, basic settings are the same in every experiment. 1. The training process is based on the Tesla P100 GPU for computation. 2. The DL models are set as pretrained before the training begins. 3. Minibatches are adopted with a batch-size of 64. 4. The L1 loss (the mean absolute error between actual and predicted values) is set as the loss function. 5. The model is trained by a stochastic gradient descent (SGD) optimizer with an initial learning rate of 0.1 and momentum of 0.9. 6. The learning rate is scheduled to be multiplied by 0.1 after every 70 epochs, and the whole training process takes 230 epochs.

**Supplementary Note S2: FEA Details**

Finite element analysis was performed using a commercial software package, ABAQUS. The resonator structures were meshed using 1^st^ order, eight-node elements with reduced integration (C3D8R). The planar dimensions of one element match the sizes of one pixel in the binary pattern. The hemispherical substrate was meshed with C3D8R elements. The size of elements in the substrate gradually transitions from small (for matching the nodes on the “anchor”) to large (for effectively mesh the large hemispherical surface) along with radial directions. The infinite element layer was meshed with one layer of CIN3D8 elements. The resonator, the substrate, and the infinite layer were all assigned the mechanical properties of polysilicon (The resonators are polysilicon structures with density$\text{ρ =2.3×}\text{10}^{\text{3}}\text{kg/}\text{m}^{\text{3}}$, Young’s modulus $\text{E=150 Gpa}$and Poisson’s ratio $\text{ν=0.29}$).

In the natural frequency analysis (with the *frequency command in ABAQUS), the fixed boundary condition was applied to the nodes on the bottom surface of “anchor”. A Subspace eigensolver was used to calculate the natural frequency, mode shape, and effective mass for each mode. The calculated, real-valued frequency of the flexural mode ($\text{ω}_{\text{flex}}$) would be plugged into the complex frequency analysis.

The FEA essence of the natural frequency analysis is numerically solving an eigenvalue problem on frequency $\omega$:

$\left( -\omega^{2}M^{MN}+K^{MN} \right)\phi_{N}=0$ (Eqn. S1)

where $M^{MN}$ is the mass matrix, $K^{MN}$ is the stiffness matrix, $\phi_{N}$is the eigenvector, and the indices $M$ and $N$ are degrees of freedom in the finite element model. In FEA, the subpsace eigensolver automatically calculates the normalized “generalized mass” associated with each mode. For any mode $\alpha$, the generalized mass associated with it $m_{\alpha}$ can be expressed as:

$m_{\alpha}=\phi_{\alpha}^{N}M^{NM}\phi_{\alpha}^{M} (no sum on \alpha)$ (Eqn. S2)

where $\phi_{\alpha}^{N}$ is the eigenvector for mode $\alpha$. The modal participation factor $\Gamma_{\alpha i}$ (for mode $\alpha$, direction $i$) describes how strongly motion along X, Y, and Z directions, or rotation about X, Y, and Z axes is represented in the eigenvector of that mode. $\Gamma_{\alpha i}$ can be expressed as:

$\Gamma_{\alpha i}=\frac{1}{m_{\alpha}}\phi_{\alpha}^{N}M^{NM}T_{i}^{M} (no sum on \alpha)$ (Eqn. S3)

where $T_{i}^{N}$ is the magnitude of the rigid body response of degree of freedom $N$ in the model to imposed rigid body motion of type $i$^42^. The effective mass $m_{\alpha i}^{eff}$ associated with mode $\alpha$, kinematic direction $i$ can be related to generalized mass and participation factor as:

$m_{\alpha i}^{eff}={(\Gamma_{\alpha i})}^{2}m_{\alpha} (no sum on \alpha)$ (Eqn. S4)

The $m_{\alpha i}^{eff}$ values can be requested as outputs of the FEA analysis, and are used for identifying each mode of interest automatically in this study.

In the complex frequency analysis, the meshes of resonator are seamlessly attached to the meshes of substrate through coincident nodes on the bottom surface of the “anchor”. The fixed boundary condition was applied only on the outermost nodes in the infinite element layer to provide the freedom for interaction between the resonator and the substrate. The complex frequency analysis took two steps in FEA. In the first step (with *frequency command in ABAQUS), a Lanczos eigensolver was used to calculate the real-valued natural frequency of the resonator + substrate combination. In the second step, a complex frequency function (with *complex frequency command in ABAQUS) was used to calculate the complex-valued frequency of the flexural mode $\text{ω}_{\text{flex}}^{\text{C}}$ and $\text{Q}_{\text{anchor}}$. In this step, the minimum and maximum frequency of interest were set as $\text{0.98*ω}_{\text{flex}}$ and $\text{1.02*ω}_{\text{flex}}$ respectively, for limiting the computation to only the mode of interest (i.e., flexural mode).

**Supplementary Figures**


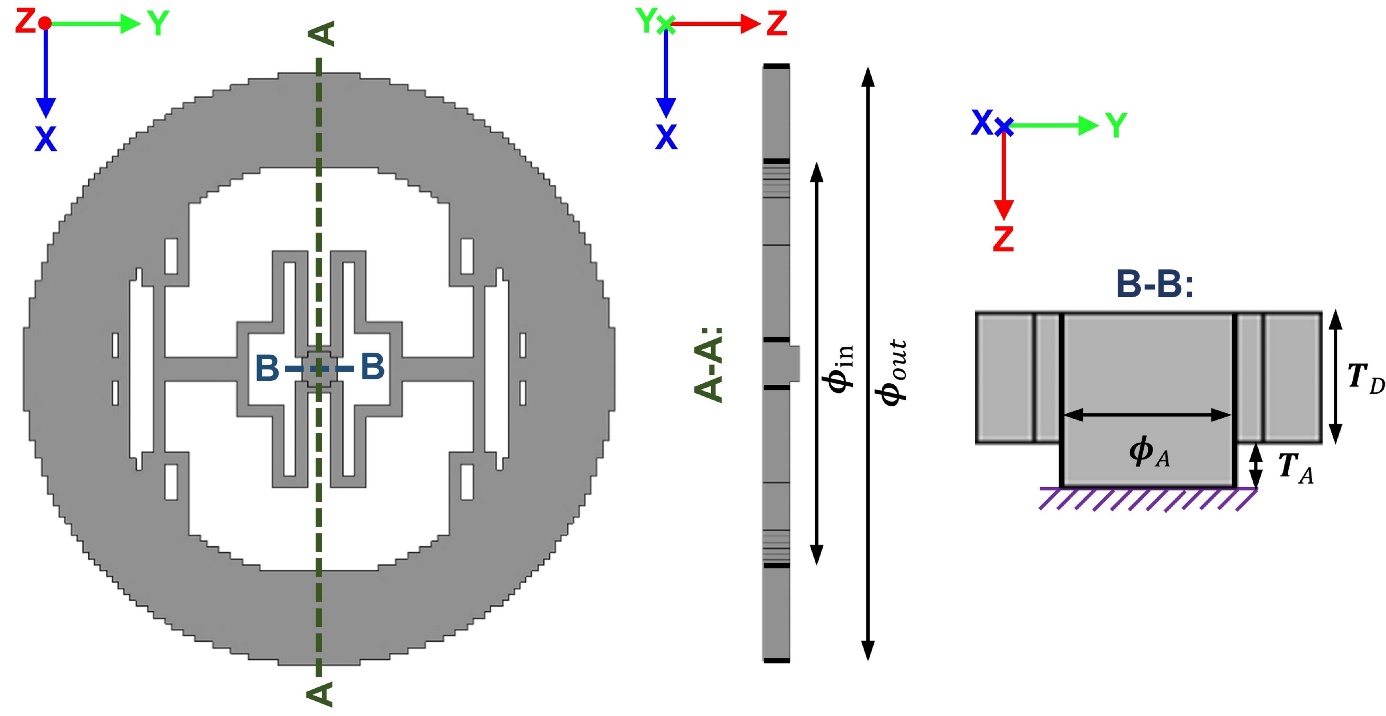


**Fig. S1** **The top, and two representative cross-sectional views of an example resonator pattern.** Dimensions: the outer ring diameter $\text{ϕ}_{\text{out}}\text{=44µm}$, inner ring diameter $\text{ϕ}_{\text{in}}\text{=30.8µm}$, stem anchor diameter $\text{ϕ}_{\text{A}}\text{= 2.64µm}$. The thickness of the disk $\text{T}_{\text{D}}\text{=2µm}$, and the height of the anchor $\text{T}_{\text{A}}\text{=0.7µm}$.


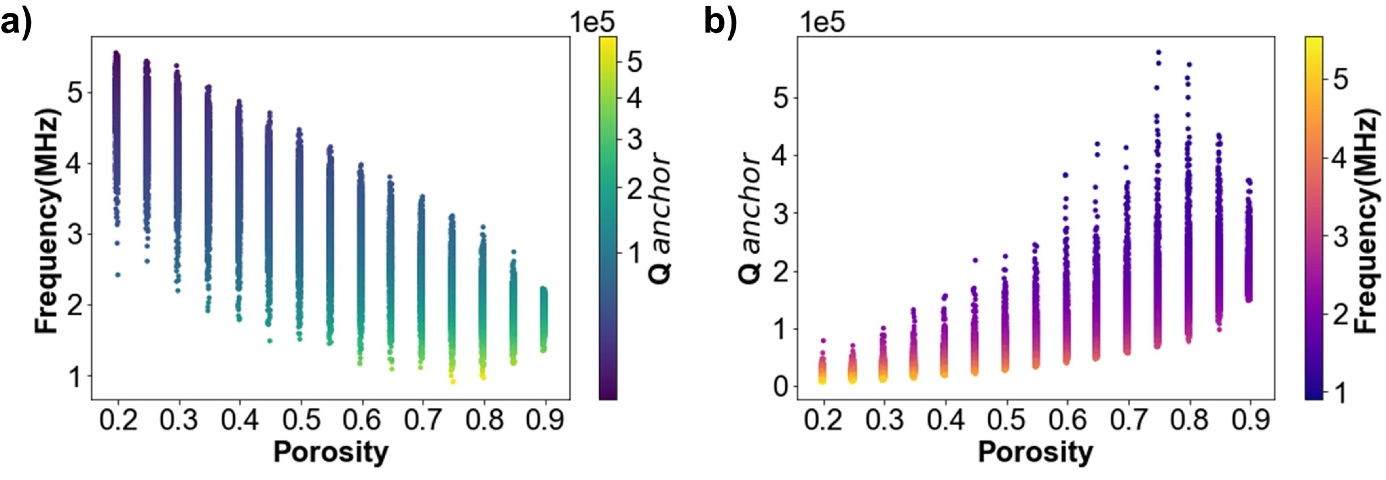


**Fig. S2**  **a** Frequency vs porosity plot, where more high-frequency samples are at low porosity and more low-frequency samples are at high porosity. Color denotes the $\text{Q}_{\text{anchor}}$ Value of each sample. **b** $\text{Q}_{\text{anchor}}$ value vs porosity, where more high $\text{Q}_{\text{anchor}}$ samples are at high porosity and more low $\text{Q}_{\text{anchor}}$ samples are at low porosity. Color denotes the Frequency of each sample.

**
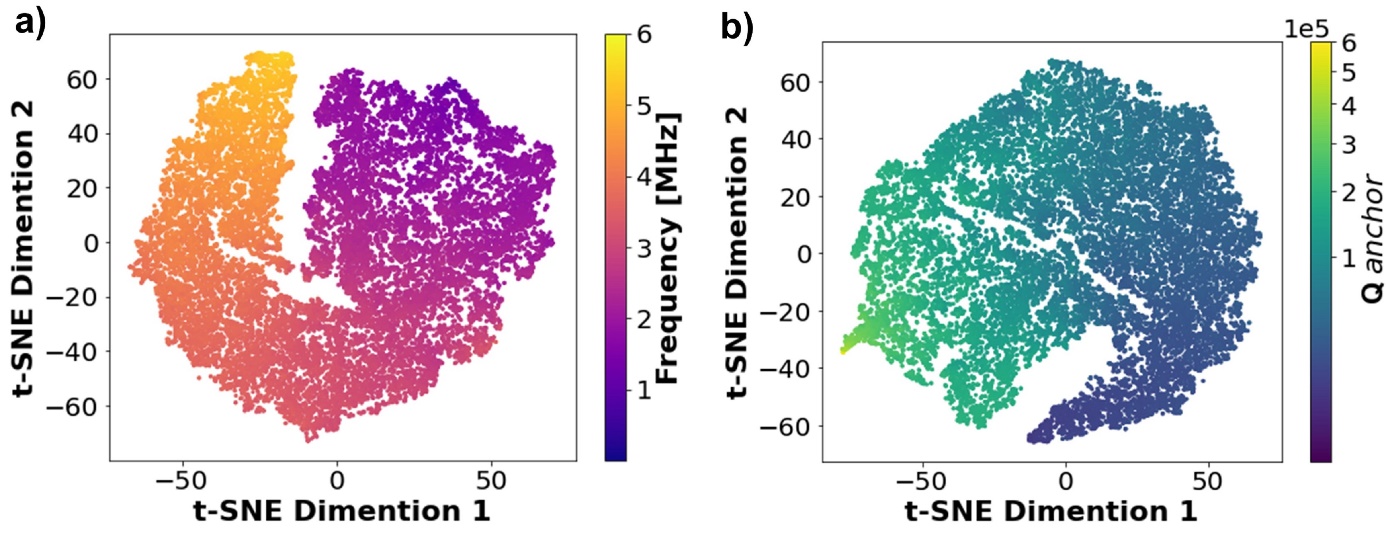
**

**Fig. S3** **Two-dimensional t-SNE analysis of the DL calculator hidden layer vectors for the training set.** Data points are colored by **a** the original frequency labels and **b** the original $\text{Q}_{\text{anchor}}$ labels.


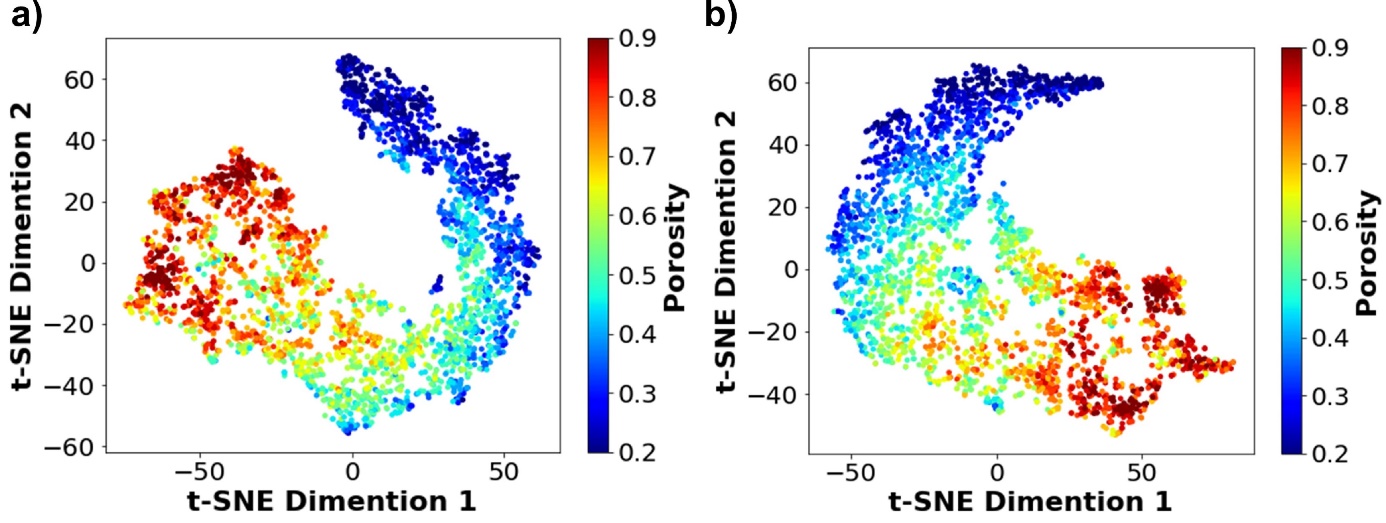


**Fig. S4** **Two-dimensional t-SNE analysis of the ML calculator hidden layer vectors for the testing set.** Data points are colored by original porosity based on the results from the **a** resonant frequency prediction model, and **b** the results from the $\text{Q}_{\text{anchor}}$ prediction model


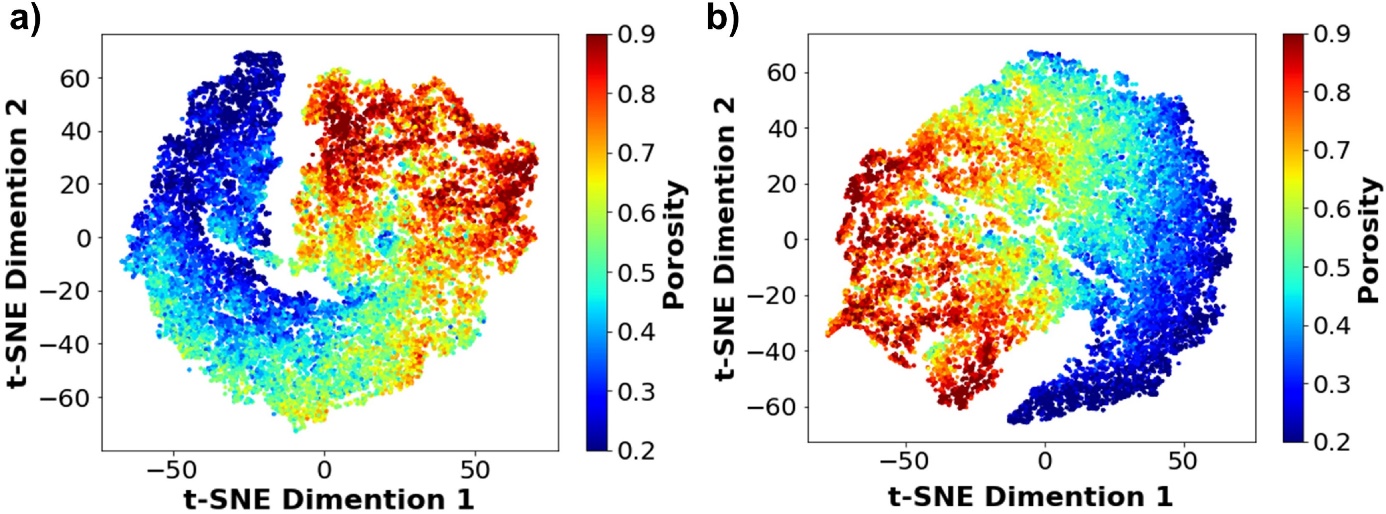


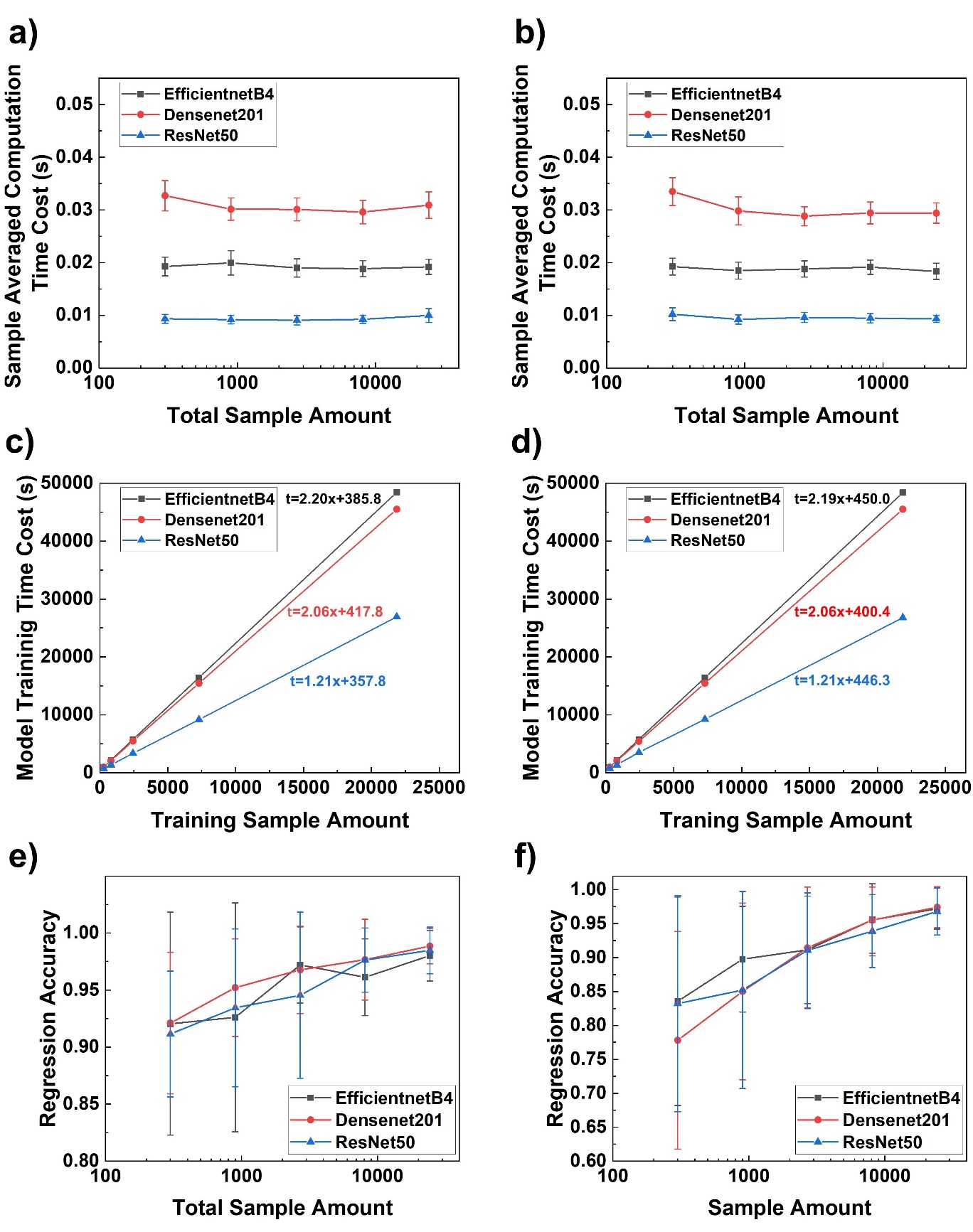
**Fig. S5** **Two-dimensional t-SNE analysis of the ML calculator hidden layer vectors for the training set.** Data points are colored by porosity based on the results from **a** the resonant frequency calculations, and **b** the results from the $\text{Q}_{\text{anchor}}$ calculations.

**Fig. S6** **The performance comparision between ResNet50 (blue), DenseNet201 (red) and EfficientNetB4 (black).** The sample averaged computation cost versus the total sample amount of the testing process for **a** frequency, and **b** $\text{Q}_{\text{anchor}}$. The total model training time cost versus the training sample amount for **c** frequency, and **d** $\text{Q}_{\text{anchor}}$, where the corresponding fitted linear equations are annotated by the same color of data points. The testing regression accuracies versus the total sample amount for **e** frequency, and **f** $\text{Q}_{\text{anchor}}$, where the three selected models showing comparable results.


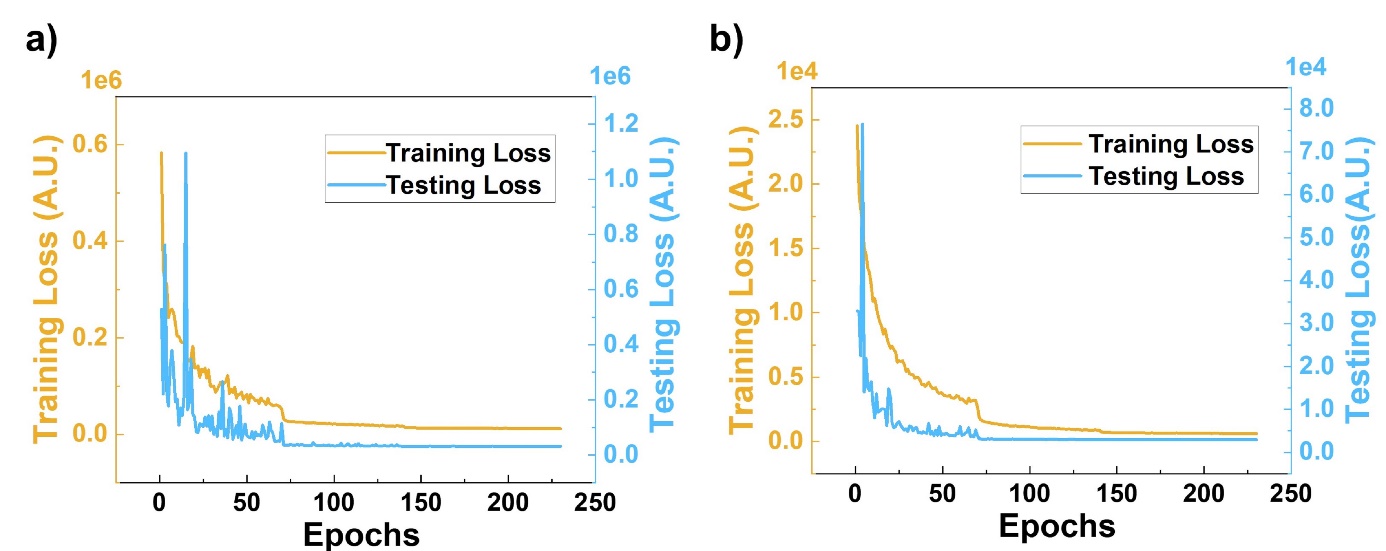


**Fig. S7 The L1 training loss and testing loss during the 230-epoch training process**. For **a** the frequency DL calculator and **b** the $\text{Q}_{\text{anchor}}$ ML calculator.


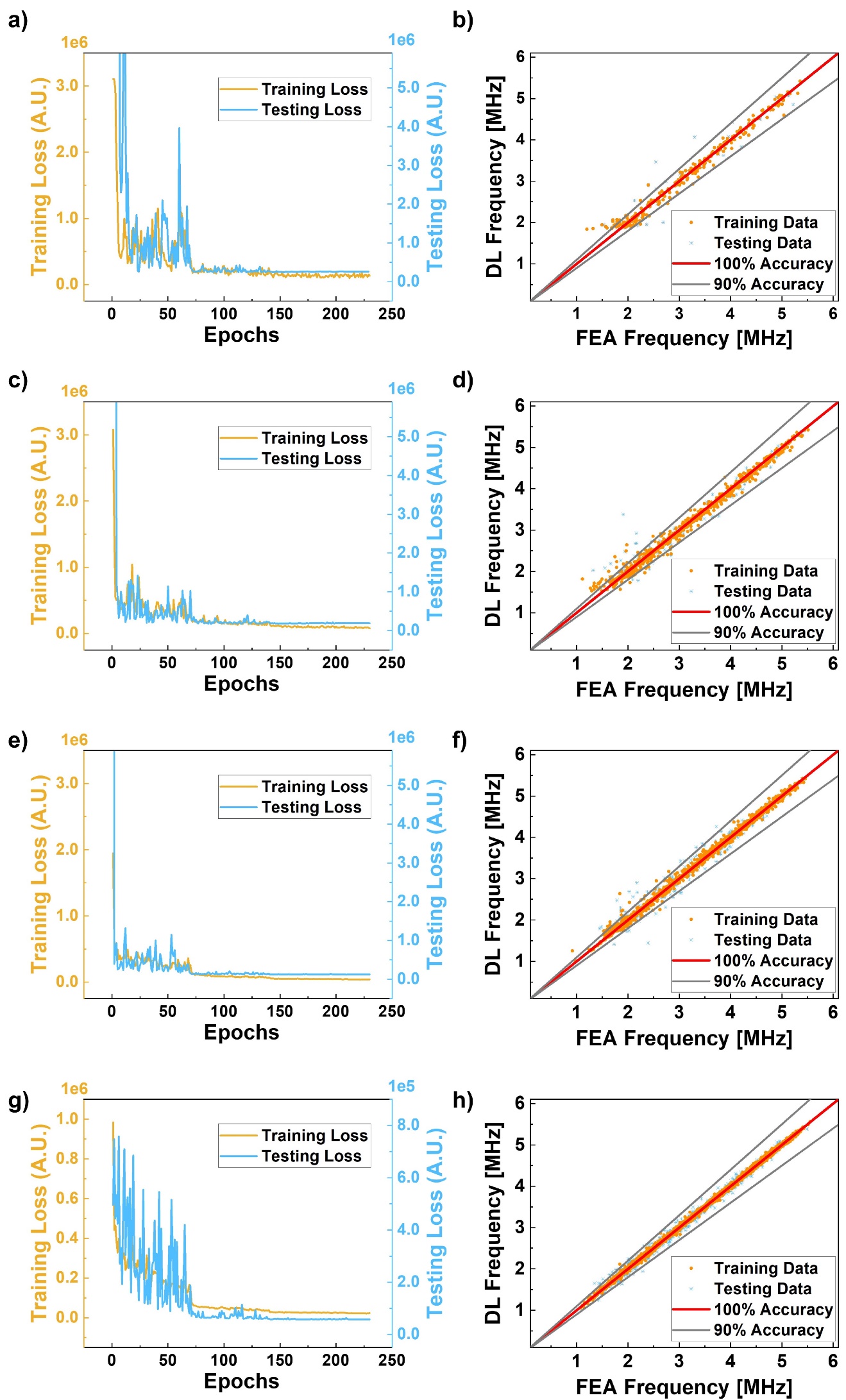


**Fig. S8** **Training and testing loss vs epochs (left column) and the comparison between DL prediction values with the FEA simulation values (right column) during the study of frequency prediction.** **a**, **b** Results with 300 datum points, with 270 training data and 30 testing data. **c, d** Results with 900 datums, with 810 training data and 90 testing data. **e,f** Results with 2700 data points, with 2430 training data and 270 testing data. **g**, **h** Results with 8100 data points, with 7290 training data and 810 testing data.


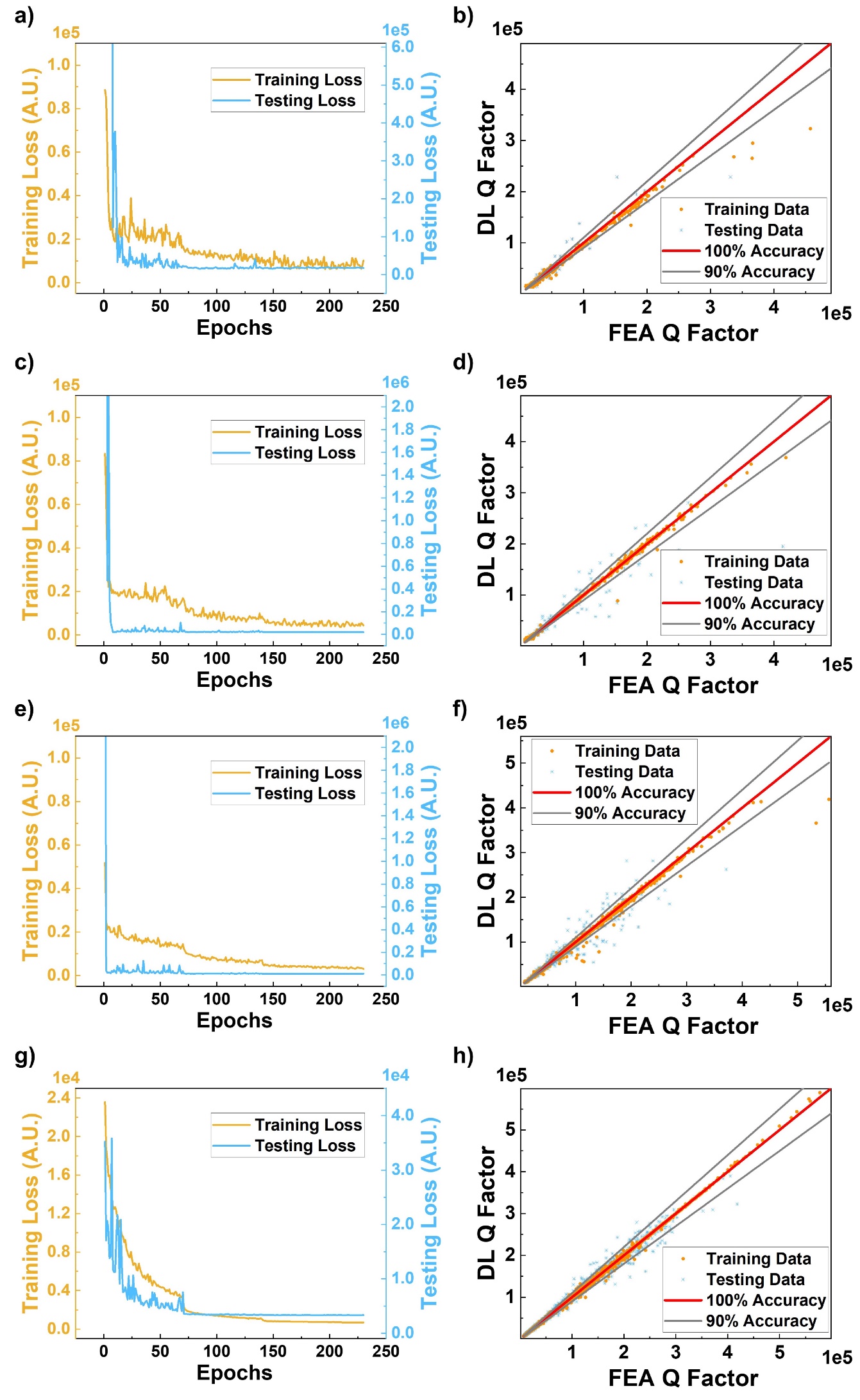


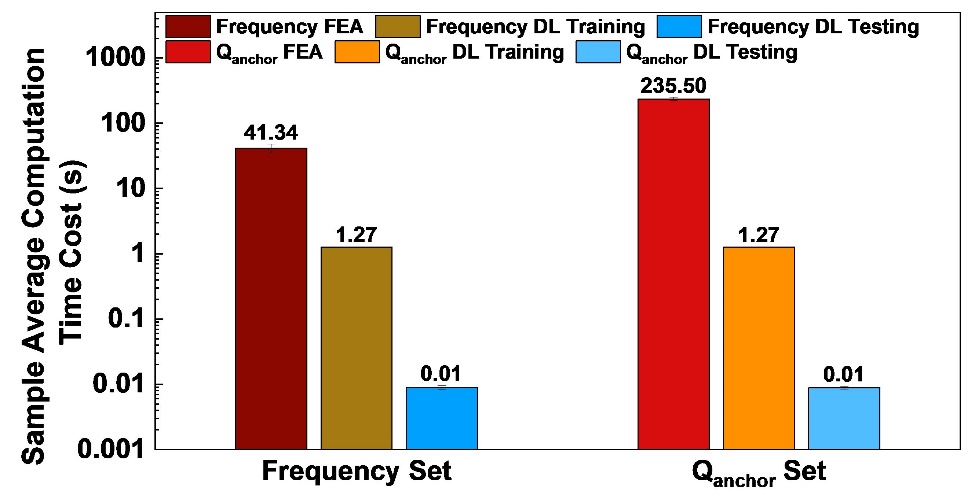
**Fig. S9** **Training and testing loss vs epochs (left column) and the comparison between DL prediction values with the FEA simulation values (right column) during the study of** $\text{Q}_{\text{anchor}}$ **prediction.** **a, b** Results with 300 datum points, with 270 training data and 30 testing data. **c, d** Results with 900 datums, with 810 training data and 90 testing data. **e**, **f** Results with 2700 data points, with 2430 training data and 270 testing data. **g**, **h** Results with 8100 data points, with 7290 training data and 810 testing data.

**
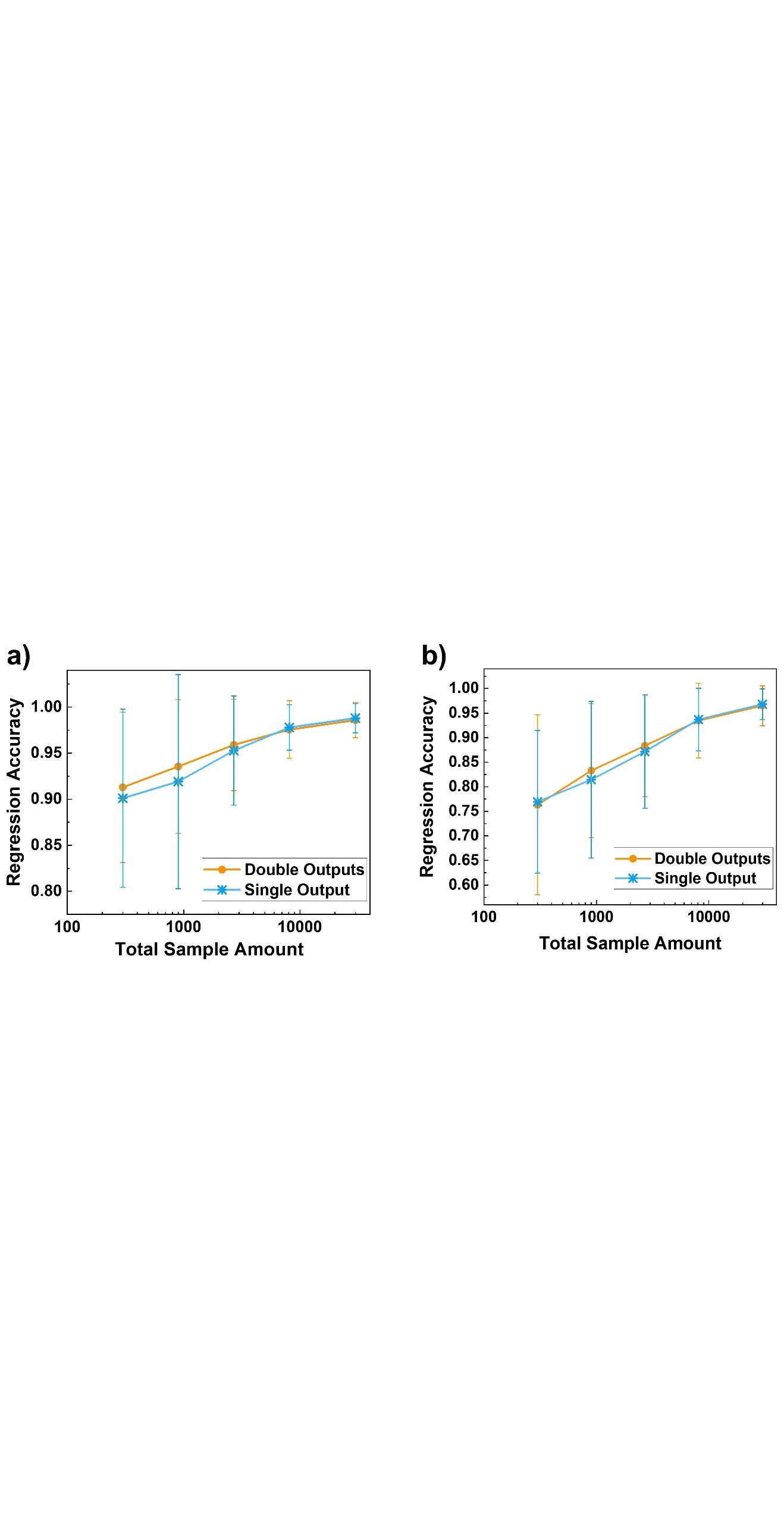
Fig. S10** **The sample averaged computation time costs of FEA simulation, single-output DL training and testing process** **for frequency (left) and** $\text{Q}_{\text{anchor}}$ **(right) respectively.**

**Fig. S11** **The testing regression accuracies of the Double-output (orange) and Single-output (blue) DL calculators versus the total sample amount.**
